# The Effect of Double – Blind Carbohydrate Ingestion during 60 km of Self-Paced Exercise in Warm Ambient Conditions

**DOI:** 10.1371/journal.pone.0104710

**Published:** 2014-08-11

**Authors:** Camila Nassif, Aline Regina Gomes, Gustavo H. C. Peixoto, Mauro Heleno Chagas, Danusa Dias Soares, Emerson Silami-Garcia, Eric J. Drinkwater, Jack Cannon, Frank E. Marino

**Affiliations:** 1 School of Human Movement Studies, Charles Sturt University, Bathurst, New South Wales, Australia; 2 School of Physical Education, Physiotherapy and Occupational Therapy, Federal University of Minas Gerais, Pampulha, Belo Horizonte, Brazil; Québec Heart and Lung Institute, Canada

## Abstract

This study evaluated double blind ingestions of placebo (PLA) versus 6% carbohydrate (CHO) either as capsules (c) or beverage (b) during 60 km self-paced cycling in the heat (32°C and 50% relative humidity). Ten well-trained males (mean ± SD: 26±3 years; 64.5±7.7 kg and 70.7±8.8 ml.kg^−1^.min^−1^ maximal oxygen consumption) completed four separate 60 km time trials (TT) punctuated by 1 km sprints (14, 29, 44, 59 km) whilst ingesting either PLA_b_ or PLA_c_ or CHO_b_ or CHO_c_. The TT was not different among treatments (PLA_b_ 130.2±11.2 min, CHO_b_ 140.5±18.1 min, PLA_c_ 143.1±29.2 min, CHO_c_ 137.3±20.1 min; *P*>0.05). Effect size (*Cohen’s d*) for time was only moderate when comparing CHO_b_ – PLA_b_ (*d* = 0.68) and PLA_b_ – PLA_c_ (*d* = 0.57) whereas all other ES were ‘trivial’ to ‘small’. Mean speed throughout the trial was significantly higher for PLA_b_ only (*P*<0.05). Power output was only different (*P*<0.05) between the sprints and low intensity efforts within and across conditions. Core and mean skin temperatures were similar among trials. We conclude that CHO ingestion is of little or no benefit as a beverage compared with placebo during 60 km TT in the heat.

## Introduction

Time to exhaustion (TTE) is extended with exogenous carbohydrate (CHO) ingestion [1]. Fluid ingestion combined with CHO has also been shown to have independent and additive benefits for exercise lasting up to 1 h [2]. More recently, a significant improvement in TTE was shown when ingesting 4–6% CHO drinks in cool (10°C) conditions, whereas, only 6% CHO was beneficial in warm (30°C) conditions [3]. Ingestion of CHO during a 40 km self-paced outdoor run increased blood glucose in comparison to a placebo (PLA) leading to improved performance [4]. As such, it is hypothesized that CHO requirement is increased during exercise heat-stress due to a shift in substrate utilization towards CHO oxidation [5].

There is also evidence suggesting PLA to be potentially confounding when testing for the effect of CHO on effort perception [6]. Thus, it is essential to account for this possibility when evaluating the effect of CHO or any other active substance, as expectations could determine the intrinsic feedback as classically shown [7]. Notably, improved exercise performance was reported when subjects believed they were ingesting CHO but were actually consuming PLA [6]. Supplementation with CHO has mainly been studied during fixed intensity protocols to volitional exhaustion. However, there is a prevailing view that these tests lack ecological validity and reliability [8], whereas, self-paced exercise has been shown to be relatively less variable and more reliable [9].

It has been shown that 100 km time trial performance was not improved with ingestion of CHO when subjects were fed, leading the authors to conclude that delaying hypoglycaemia by increasing pre-exercise liver glycogen stores could offset any potential benefits of CHO ingestion during exercise [10]. Alternatively, these authors also raised the possibility that the ergogenic benefit of carbohydrate loading could be related to the placebo effect. Many studies have shown carbohydrate supplementation to improve exercise performance. However, this view is highly dependent on the performance parameters. For example, when comparing a carbohydrate – electrolyte drink with a placebo, cycling performance in warm (26.6–27.7°C, 67 to 68% relative humidity, *rh*) conditions was indeed improved, but the performance criteria were predetermined so that subjects were required to perform a pre-calculated amount of work. Similarly, in a 40 km run, carbohydrate – electrolyte replacement did not improve performance until after 35 km was achieved with only six of the eight subjects completing the test with heart rate, core temperature and ratings of perceived exertion (RPE) all being similar between carbohydrate-electrolyte and placebo conditions [4]. As far as we are aware there are no studies reporting the effect of CHO ingestion during long duration self-paced cycling in the heat.

Therefore, the purpose of this study was to test the effect of a 6% CHO beverage or an equivalent 6% CHO capsule versus corresponding PLA trials during a 60 km self-paced time trial in the heat. A further aim was to examine if any changes in performance between trials were associated with a pacing strategy.

## Materials and Methods

### Participants

Ten well trained competitive males volunteered to participate in the study (age 26±3 years, body mass 64.5±7.7 kg, stature 170±9 cm, peak power output (PPO) 323±41 W, maximal O_2_ consumption (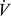
O_2max_) 70.7±8.8 ml.kg^−1^.min^−1^) having completed a health screening questionnaire and released by a physician. Approval for the study was obtained from the Ethics in Human Research Committee of Charles Sturt University and each subject signed a letter of informed consent.

Preliminary measurements included skinfolds (biceps, triceps, chest, sub axillary, subscapular, suprailiac, abdominal, thigh and calf), 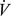
O_2max_ and PPO. A BIOPAC Systems (Goleta, CA, U.S.A.) spirometer was used to determine expired gas concentration during a progressive 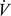
O_2max_ testing protocol as previously described (Kay et al., 2001). The progressive test commenced at 100 W with 10 W increments every 30 s until voluntary termination. The 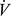
O_2max_ test was performed in ambient conditions of 22±1°C and 68±6% relative humidity (*Rh*). Participants cycled with their own bicycle attached to a Flow Trainer (TACX, T1684, Netherlands). Throughout the test, participants cycled in a seated position but were permitted to change gears and/or cadence throughout the test, provided that the required power increments were maintained at all times.

### Experimental Design

Each subject completed four randomized double-blind experimental trials. Subjects ingested either a PLA or 6% CHO across four trials using the following modes of ingestion; either as a beverage (PLA_b_ and CHO_b_) or in capsules (PLA_c_ and CHO_c_) with distilled water. Subjects were informed that CHO was to be ingested for all treatments. Trials were coded and prepared by an individual not directly involved with the study so that conditions were only revealed to the investigators at the completion of the study. Trials were performed at the same time and day of each week but separated by 12±7 days to minimize motivational differences, diurnal variation and training effects. Participants were required to maintain usual training routine but abstain from training, strenuous activity, caffeine and alcohol consumption for at least 24 h prior to all trials.

### Pre-testing procedures and requirements

Two days prior to each trial, subjects were required to follow a diet with 60 to 70% CHO, 1.2 to 1.7 g of protein/kg of body mass, and 20 to 25% fats, as individually prescribed by a dietician. This procedure maximised recovery of muscle glycogen stores following the last day of training prior to each trial. Subjects were not required to fast overnight prior to each trial but were required to consume breakfast on the morning of each trial as prescribed by the dietician.

### Time trial protocol

Subjects mounted the cycle and commenced a warm-up at 50% of PPO. Following the warm-up, EMG electrodes were attached to the quadriceps muscle of their dominant leg (described subsequently). Each participant completed four trials in each of the different conditions (PLA_b_, CHO_b_, PLA_c_, CHO_c_), consisting of a 60 km self-paced time trial (slope+2, TACX^®^program) punctuated with 4×1 km sprints (high intensity, HI) at 14, 29, 44 and 59 km. When not sprinting, subjects were required to complete the time between sprints as quickly as possible (low intensity efforts, LI). Subjects were encouraged to complete each trial as quickly as possible, were permitted to alter gear ratio and cadence and pedal while standing when they desired, other than during the sprints and when EMG data were collected which required a seated position to prevent changes in muscle fibre recruitment patterns that can occur with postural changes. All trials took place in an environmental chamber (Russels Technical Products, model WMD-1150-5, Holland, MI, U.S.A.) in a warm environment (32°C, 50% *Rh*). A fan was placed in front of the subject, positioned towards the head and torso when in a normal cycling position with a wind speed of 3 m/s, with the naturally circulating air provided by the chamber during exercise. The test was terminated if one or a combination of the following criteria were achieved; 1) core temperature reached 39.5°C, 2) RPE of 20, 3) the subject terminated exercise due to volitional fatigue, and/or 4) any sign of illness or discomfort by the subject. Participants received feedback on distance completed at 3 km intervals throughout each trial and at the start and finish of each sprint, but no other performance or physiological feedback was made available to subjects until the study was completed.

Fluids were ingested at 5 km, after each sprint (15, 30, 45 km) and at 55 km. The fluid temperature was 4±1°C. Each subject received 284±30 ml of distilled water and capsules (4 ml.kg^−1^ of body mass) or beverage depending on the trial (described subsequently) with all hydration procedures conducted as previously described [11]–[14]. The amount of CHO and PLA powder in capsules was calculated to match the volume of water so that the mixture of water/powder or water/capsules corresponded to a 6% CHO solution. Whey protein was chosen as a bulking powder and as PLA given that administration of amino acids during exercise and in combination with CHO have been shown not to provide any metabolic contribution to endurance exercise for at least 2 h either post ingestion or when ingesting during exercise [1], [15], [16]. The powder was prepared by a commercial chemist (Stenlake, Sydney, NSW, Australia) who guaranteed matching taste, colour, flavour and purity.

### Physiological measures

Hydration status was assessed by urine specific gravity (USG) (503 Nippon Optical Works, Japan) before and at the completion of exercise. Blood samples were taken from the middle digit of the hand at rest, 5 km intervals throughout each time trial and immediately post exercise for the determination of blood lactate concentration [La^−^] (Lactate Pro, ARKRAY, Kyoto, Japan) and glucose concentrations [Glu] (Accu-check Advantage, Roche Diagnostics Corporation, Indianapolis, USA). Heart rate was continuously monitored (Polar Electro OY M61, Kempele, Finland) and recorded at 2 km intervals. The RPE [2], [17] was recorded every 5 km, before and after each sprint and when EMG was recorded.

Core temperature (*T*_c_) was measured using the CoreTemp Telemetry System (HQInc. Wireless Sensing System and Design, Palmetto, Florida, USA). Participants ingested the telemetry pill the night before the trial and were advised by the researcher at what time it should be ingested according to the participants bowel motion. Skin temperature was monitored with skin thermistors attached at four sites (chest, arm, thigh, leg) and connected to a digital telethermometer (Zencor/Zentemp 5000, Victoria, Australia) and recorded every 2 km. Mean skin temperature (*T*_sk_) was calculated as previously described [3], [18].

### Surface electromyography (EMG)

Surface EMG was sampled during cycling using single-differential pre-amplified EMG electrodes with bar configuration of 1 mm×10 mm, an inter-electrode distance of 10 mm and bandwidth of 20–450 Hz (DE-2.1, Delsys, Boston, MA, USA) linked to an amplifier where signals where acquired at a gain of 1000 V/V during cycling with common mode rejection ratio>90 dB (Bagnoli-4, Delsys, Boston, MA, USA). EMG electrodes were attached half way between the superior anterior iliac spine and the superior part of the patella for rectus femoris (RF) and at the distal 4/5 point between the superior anterior iliac spine and the joint space in front of the anterior border of the medial ligament at the knee for vastus medialis (VM). Skin was prepared by shaving, abrading of the outer layer of epidermal cells, and removing of oil and dirt using an alcohol swab. A disposable gel adhesive electrode was used as a reference (#2330, 3 M, Red Dot, St. Paul, MN, USA) positioned on the acromion process of the right shoulder.

### Cycling EMG

EMG signals from RF and VM were recorded for the first 30 s of each LI effort at 7.5, 21.5, 36.5 and 51.5 km and throughout each sprint at 14, 29, 44 and 59 km. EMG signals from the amplifier were fed to a host computer for A/D conversion at 16-bit resolution (DAQCard-6036E, National Instruments, Austin TX, USA) and sampled using EMGWorks Signal Acquisition software (version 3.0, Delsys, Boston, MA, USA) at a rate of 2000 Hz. For analysis of the LI effort, the entire 30 s of EMG data were used while during the sprints the middle 30 s of the data set were used. EMG signals were high-pass filtered using a second order Butterworth filter at 20 Hz to remove movement artefact and the linear envelope computed as the root-mean-square (RMS) using a sliding window of 125 ms with a step of 62.5 ms. EMG signals were then quantified by summating the RMS EMG signals from both muscles then calculating the area under the curve (iEMG). The iEMG for each LI effort and sprint were then normalised against the data obtained during the first LI effort.

### Statistical analysis

Data analyses were performed by repeated measures ANOVA using GraphPad Prism 6.0. The source of significance was located using Tukey’s HSD post-hoc tests. All data are presented as means ± SD with significance set at *P*<0.05. Effect sizes (ES, *Cohen’s d*) were calculated to analyse the potential trends in time and speed for respective conditions. An ES of<0.2 is classified ‘trivial’, 0.2–0.5 as ‘small’, 0.5–0.8 as ‘moderate’ and>0.8 as ‘large’ effect.

## Results

### Time trial, power output and EMG

**Figure 1:**
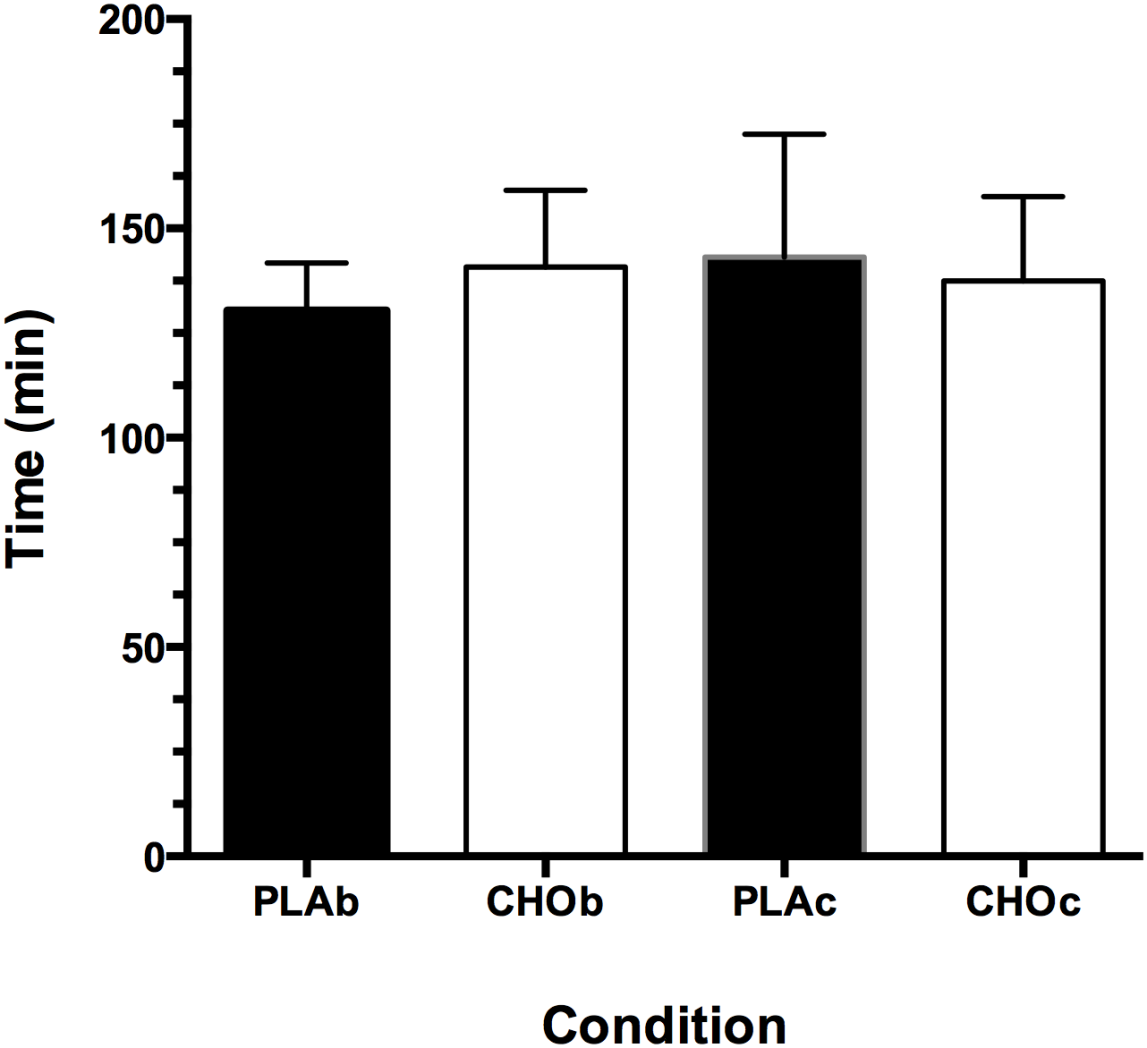
Time to complete the 60 km time trial for each condition. PLA is placebo and CHO is carbohydrate; b and c denote beverage and capsule conditions, respectively.

The times to complete the 60 km TT were not different among conditions (*P* = 0.32; see Figure 1). The ES for each of the combination of PLA and CHO trials are shown in Table 1. The ES for time taken to complete 60 km ranged from 0.1–0.7 indicating ‘trivial’ to ‘moderate’ effect amongst conditions.

**Table 1:** Effects sizes (*Cohen’s d*) for each of the combinations of conditions.

Condition	Effect Size (*Cohen’s d*)	Classification	*P* value
PLAb – CHOb	0.68	Moderate	.08
PLAb – PLAc	0.57	Moderate	.20
PLAb – CHOc	0.43	Small	.24
PLAc – CHOcCHOb – CHOcCHOb – PLAc	0.220.17	Small	.30
	0.10	Trivial	.60
		Trivial	.81

Effect sizes <0.2 ‘trivial’; 0.2–0.5 ‘small’; 0.5–0.8 ‘moderate’ and >0.8 ‘large’.

PLA is placebo and CHO is carbohydrate; b and c denote beverage and capsule conditions, respectively.

**Figure 2:**
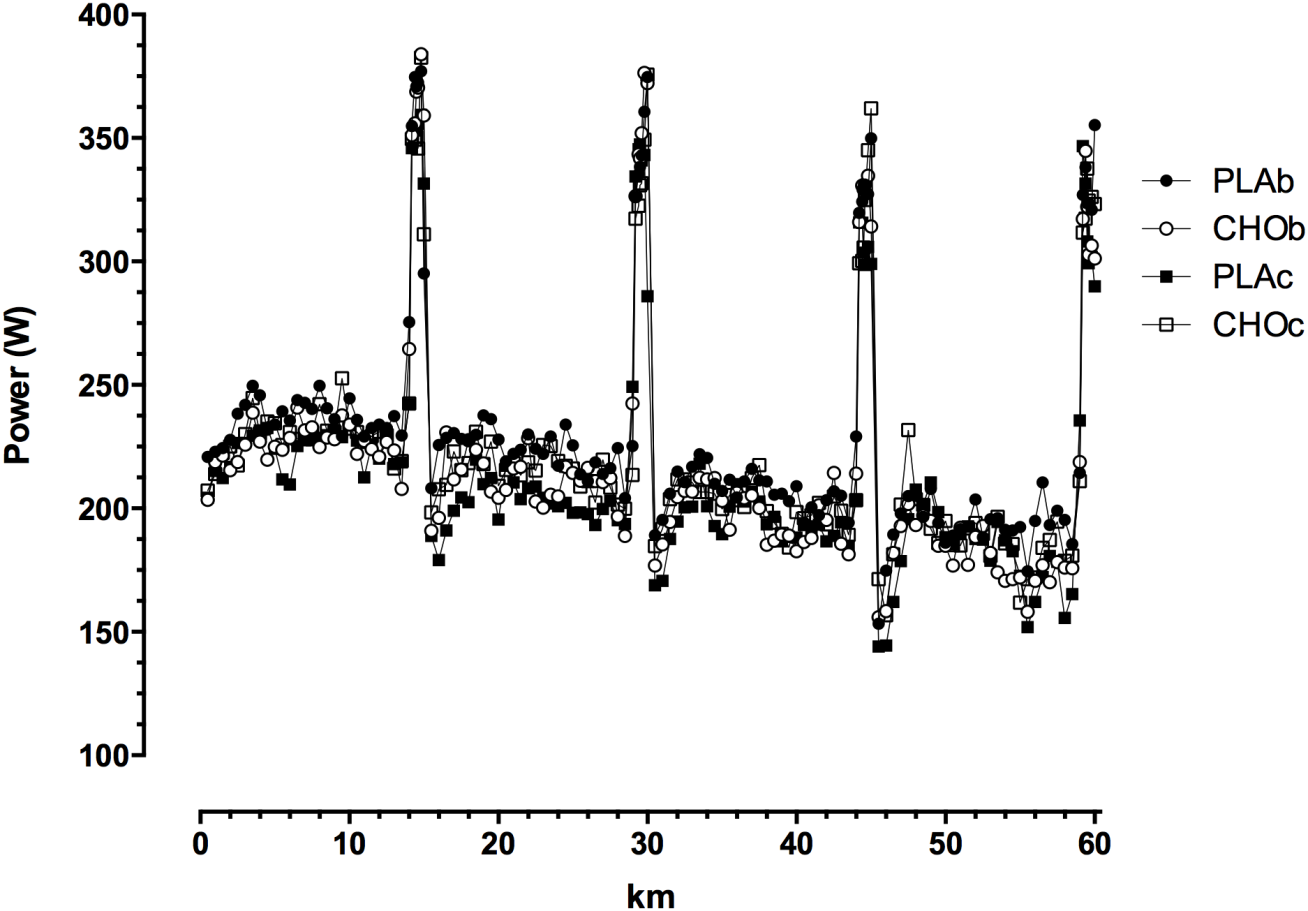
Power at each 0.5 km interval over the 60 km time trial for each condition. PLA is placebo and CHO is carbohydrate; b and c denote beverage and capsule conditions, respectively.

**Figure 3:**
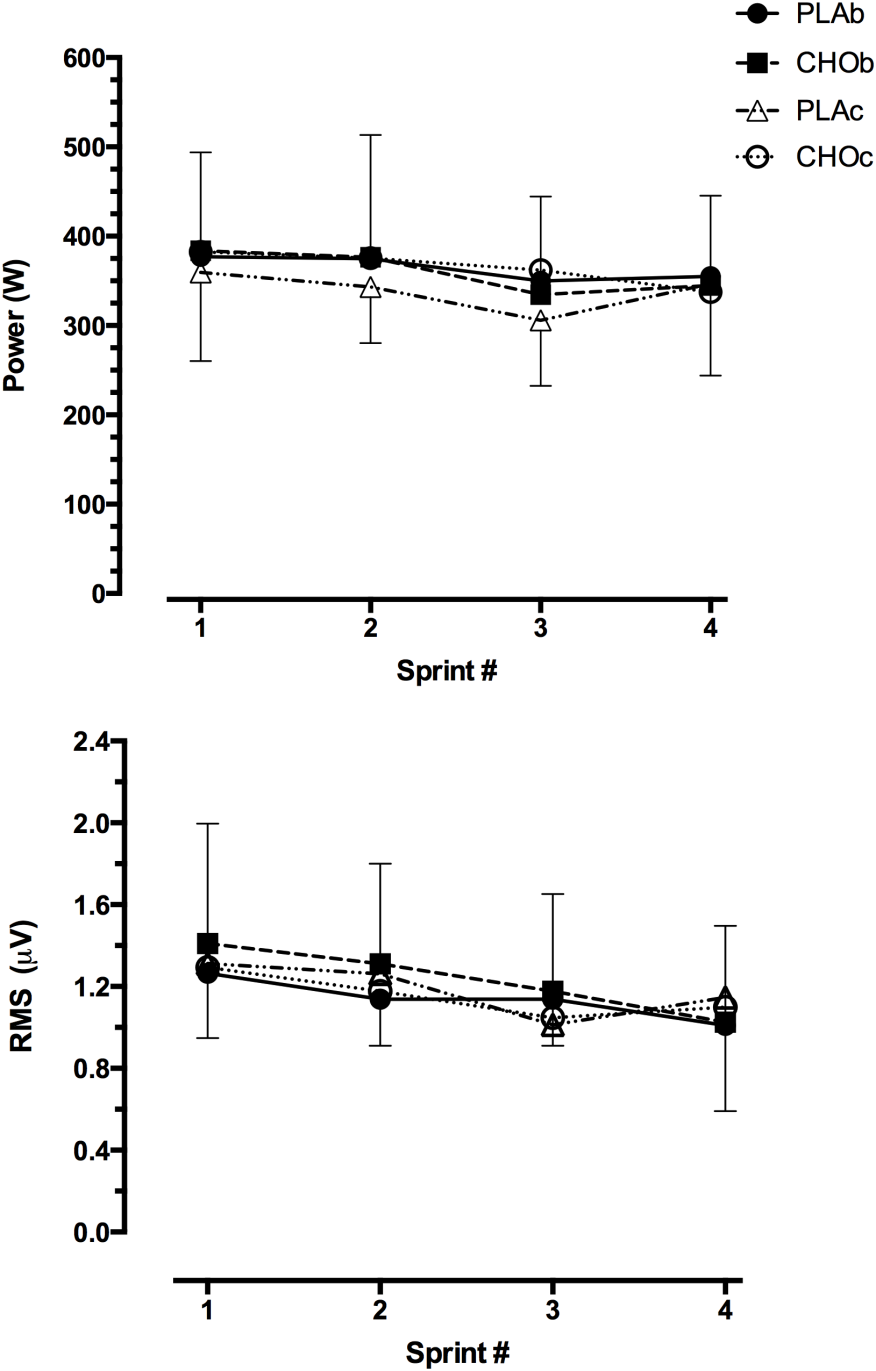
Power (top) and RMS (bottom) for each of the 1 km sprints for each condition. PLA is placebo and CHO is carbohydrate; b and c denote beverage and capsule conditions, respectively.

Power outputs at 0.5 km intervals over the 60 km are shown in Figure 2 for each condition. Power outputs were consistently higher (*P*<0.05) during the sprint sections compared with LI efforts across all trials. On average across the four trials, power output during the LI efforts was 207.0±6.4 W compared with 334.9±7.2 W (*P*<0.001) during the 1 km sprints. The power outputs for each sprint with the relative RMS at each of the 4×1 km sprints are shown in Figure 3. There were no differences in power either between sprints or conditions. Although there was a tendency for RMS to decrease from the initial sprint to the last sprint for each trial, the RMS was not statistically different either between sprints or conditions. Table 2 shows the mean speed over the trial for each of the trial sections (1 km sprints and LI). The mean speed during PLAb was higher (*P*<0.05) compared with the other conditions. However, this difference during PLAb did not translate to differences over the 1 km sprints or the LI efforts among conditions. The ES for mean speed ranged from 0.06–0.20 indicating a ‘trivial’ effects. In each condition the speed during the 1 km sprints was significantly (*P*<0.0001) higher than the LI efforts.

**Table 2:** Mean speed (km/h) over the 60 km time trial for each condition and in the different sections of the trial.

	Mean Speed	1 km Sprints	Low Intensity Efforts
PLAb	30.23±5.70*	39.46±1.52#	27.92±3.68
CHOb	29.46±6.25	39.49±2.35#	26.86±3.75
PLAc	29.11±5.97	38.78±2.16#	26.47±4.36
CHOc	29.72±5.36	38.39±1.18#	27.45±3.88

PLA is placebo and CHO is carbohydrate; b and c denote beverage and capsule conditions, respectively.

*P<0.05 compared with conditions;

# *P*<0.0001 compared with Low-Intensity efforts.

### Hydration and metabolic responses

Although differences in USG were observed between pre (range 1.010–1.012) and post exercise (range 1.016–1.019), these data indicate that subjects commenced and terminated each trial in a hydrated state as USG was<1.029 in all cases. The [Glu] was higher in both CHO_b_ and CHO_c_ trials compared with PLA during and at the end of the time trial. The mean [Glu] was significantly (*P*<0.0001) higher for the CHOb and CHOc trials at 5.5 and 5.6 mmol.l^−1^ respectively, compared with PLAb and PLAc of 5.3 and 5.0 mmol.l^−1^, respectively. No differences in [La^−^] were observed among trials (*P*>0.05). Mean pre-exercise [La^−^] was 1.7±0.6 mmol.l^−1^ which increased to 2.5±1.3 mmol.l^−1^ during the LI efforts and then to 6.9±2.2 mmol.l^−1^ during the sprints. Subjects finished trials with [La^−^] values of ∼5.9±1.9 mmol.l^−1^.

### Heart rate and RPE

The HR response was not different among trials (*P*>0.05). HR increased significantly during the sprints compared with the LI efforts (*P*<0.05). Subjects started exercise with an average HR of 71 beats.min^−1^ increasing to ∼157 beats.min^−1^ during the LI efforts and to ∼181 beats.min^−1^ during the sprints, finishing 60 km with a HR of ∼179 beats.min^−1^. The average HR during the 60 km time trial was ∼160 beats.min^−1^. RPE was not different between trials, however, as expected RPE increased during the sprints compared with the LI efforts. The average RPE for each time point was 13±3 during LI and 16±3 during sprints, reaching 17±3 at the end of the trials with a mean RPE of 14±3 during the 60 km.

### Thermoregulatory responses

Trials commenced with similar *T*_c_ (36.9–37.1°C; *P*>0.05), terminating exercise with a *T*_c_ of 38.1±0.5°C (*P*<0.05). The *T*_c_ was maintained at 38.0±0.5°C throughout exercise. The corresponding *T*_s_ was not different between trials commencing exercise at ∼32.0°C, increasing to 34.0±0.6°C (*P*<0.05) over the 60 km.

## Discussion

We have previously shown that ingestion of CHO either as capsule or beverage did not improve TTE in the heat unless both the subject and the researchers had knowledge of the capsule contents [4], [19]. In that study we concluded this was likely due to the influence and combined effect of CHO ingestion and knowledge of what was actually ingested. We extend these previous findings with the novel result showing that independent of CHO ingestion method; capsule or beverage, 60 km self-paced cycling time trial in the heat did not improve with the ingestion of carbohydrate. These findings suggest that CHO in the form of a beverage does not provide any performance benefit over a PLA beverage or capsule. Rather, the PLAb was moderately effective over the PLAc. In addition, the data in Table 2 show that a higher mean speed was achieved over the 60 km with PLAb, indicating a performance benefit over the CHO beverage and capsule. These combined findings suggest that the masking achieved by the capsule raises the uncertainty of the contents and performance is not significantly altered when ingesting capsules. We can only conclude this to be the case since the other ES for the combination of trials ranged from ‘small’ to ‘trivial’ (Table 1). The overall findings for time to complete the 60 km in the heat suggests that CHO as a beverage has no significant effect over a capsule or PLA.

Our findings are in contrast with previous research reporting enhanced performance with the ingestion of a CHO beverage [4], [20]–[22]. Although our negative finding is somewhat difficult to reconcile, we can suggest the following possibilities. First, our method of serial ingestion of fluids and capsules is relatively unique with only few others undertaking a similar protocol [19], [23] making direct comparisons with other studies difficult. Second, our subjects commenced their respective trials in a fed state which might have diminished the potential effect of exogenous CHO intake during exercise as previously shown [23]. In fact, these authors showed that ingestion of CHO in capsule form elevated blood [Glu] compared with PLA, but this did not translate to increased time to fatigue whilst cycling at 66% VO_2peak_ in 22°C and 88% *rh*. A third possibility which has recently been proposed is the lack of sustainable effect of serial CHO mouth rinsing in cycle sprinting performance [24]. These authors found that CHO mouth rise only improved power output over the initial 5 s of a 30 s sprint, concluding that the improvement may come at a greater relative cost for the remainder of the sprint. Thus, as the sprints in the present study were 1 km distances, it is also likely that any immediate oral effect of the CHO beverage was compromised due to the longer sprinting period. This might also partly explain why the CHOb and CHOc had similar trivial effects (see Table 1 & 2).

It has also been shown that a CHO mouth rinse can influence performance or muscle activation [6], [14] suggesting that oral and pharyngeal receptors could be activated when glucose is available in the mouth. The present study shows that independent of subjects being able to taste the beverages, endurance performance was moderately improved in the heat only when compared with the placebo beverage.

In agreement with previous findings [3], [25] the [Glu] in the present study increased during CHO compared with PLA. Therefore, we can only conclude that the magnitude of the changes in [Glu] in the present study did not influence exercise performance, suggesting that this parameter may not be critical during long duration self-paced exercise in the heat. In a previous study where a total of ∼75 g of branched chain amino acids (BCAA; 50% valine, 35% leucine, 15% isoleucine) were administered before and during a 100 km time trial (∼2.7 h), no effects on either metabolism or performance were observed when BCAA were combined with a 5% CHO solution compared with CHO alone [9], [16]. Therefore, it is unlikely that the constituent whey powder in the present study had additive metabolic value given the performance time ranging from 2.4–2.2 h. Similarly, when administering 6% CHO as capsules at 15 min intervals during TTE, there were no performance differences were between CHO (∼92 min) and PLA (∼93 min) despite a higher [Glu] with CHO [23]. As expected, [La^−^] corresponded to the intensity of exercise with the highest concentrations occurring during 1 km sprints, whilst these concentrations decreased during the LI stages. Since [La^−^] and HR were not different between trials and values were always similar at each time point, indicates that subjects at least gave an equivalent effort at each stage of the trial. Since subjects in our study were well-trained competitive athletes, our findings and those of others suggest that individuals competing in events lasting up to 2 h are not likely to gain an advantage by supplementing with CHO during exercise.

In the present study there was a similar RPE at the end of exercise independent of what was ingested. The prescribed diet was designed to minimise variability in pre-exercise intramuscular glycogen, and although not confirmed by muscle biopsy, it is reasonable to expect that glycogen stores were similar with dietary (high CHO) and exercise control [6]. However, if glycogen stores were different in any of the trials a difference in exercise performance should have been apparent, but this was not the case. As it is thought that the highest RPE will likely be achieved before complete depletion of the energy substrate, there could be a central anticipatory mechanism influencing the maximal RPE terminating exercise before this maximal value is ever attained [26], [27].

Participants commenced and finished 60 km in a hydrated state (as assessed by USG), so that hydration could be one reason for the moderate to trivial differences observed in performance. Nevertheless, we have previously observed that enough fluid ingestion preventing changes in body mass does not improve performance compared with restricted fluids over 60 min of self-paced exercise in the heat [28]. As *T*_c_ responses were similar across trials, it is unlikely that CHO had an effect on this variable. Although a more pronounced rise in *T*_c_ would be expected over ∼2.3 h trial, this might have been the case if the exercise was set at a fixed intensity. A recent study evaluating the effect of CHO (0–6%) on TTE in both cool (10°C) and warm (30°C) conditions report *T*_c_ at exhaustion to be no higher than ∼38.5°C in the heat even when TTE was significantly reduced [3]. Perhaps in a self-paced trial the ability to alter moment-by-moment muscle force and power outputs, and presumably the metabolic heat production so that a maximal level of heat accumulation is never attained [29]–[31], produced similar *T*_c_ responses across conditions.

As impaired exercise performance in the heat is thought to be related to a reduction in muscle recruitment [32], EMG was measured at different trial stages. The RMS (Fig. 3) generally tracked the power output during the LI and 1 km sprints. This result suggests that neuromuscular activity was not influenced by what was or even thought to be ingested. The most salient result is that muscle recruitment was almost identical at the end of the 60 km for all trials [33]. The fact that pacing, as measured by speed and power output were similar at these distances along with peaks in heart rate, indicates that either these measures are less sensitive than the measure of muscle recruitment or that muscle recruitment is a tightly controlled response occurring well ahead of the required exercise end-point.

In conclusion, as difference in time to complete the 60 km time trial in the heat was not improved with ingestion of CHO as either beverage or capsule compared with PLA, it appears that given the opportunity to self-pace over a long duration, exogenous carbohydrate availability might play little or no role in improving performance in fed subjects.
